# Systematic review and meta-analysis of augmented reality in medicine, retail, and games

**DOI:** 10.1186/s42492-020-00057-7

**Published:** 2020-09-16

**Authors:** Pranav Parekh, Shireen Patel, Nivedita Patel, Manan Shah

**Affiliations:** 1 Department of Computer Engineering, Nirma University, Ahmedabad, Gujarat 382481 India; 2 Department of Chemical Engineering, School of Technology, Pandit Deendayal Petroleum University, Gandhinagar, Gujarat India

**Keywords:** Augmented reality, Software, Medicine, Gaming

## Abstract

This paper presents a detailed review of the applications of augmented reality (AR) in three important fields where AR use is currently increasing. The objective of this study is to highlight how AR improves and enhances the user experience in entertainment, medicine, and retail. The authors briefly introduce the topic of AR and discuss its differences from virtual reality. They also explain the software and hardware technologies required for implementing an AR system and the different types of displays required for enhancing the user experience. The growth of AR in markets is also briefly discussed. In the three sections of the paper, the applications of AR are discussed. The use of AR in multiplayer gaming, computer games, broadcasting, and multimedia videos, as an aspect of entertainment and gaming is highlighted. AR in medicine involves the use of AR in medical healing, medical training, medical teaching, surgery, and post-medical treatment. AR in retail was discussed in terms of its uses in advertisement, marketing, fashion retail, and online shopping. The authors concluded the paper by detailing the future use of AR and its advantages and disadvantages in the current scenario.

## Introduction

Significant advances in technology in contemporary times have made many things possible such as creating virtual worlds or enhancing existing real-world objects and scenarios through multiple sensory modes [1]. Augmented reality (AR) and virtual reality (VR) have the capability to alter the way entertainment, shopping, health activities, recreation, etc. are perceived [2]. Although VR and AR are often assumed to be the same, they are considerably different. AR, also termed mixed reality [3, 4], is a mapping of virtual objects onto the real world whose elements are augmented using sensory inputs. VR is a complete immersion in an artificial environment created using software [5]. This environment is presented to and accepted by the user as a real environment. This difference forms the basis of the functioning of virtual technology. Both AR and VR are often combined to attain specific goals [6].

AR, which was commercialized long ago, has played a major role in reshaping the existing manners of performing activities. However, owing to certain challenges, the technology did not achieve the expected results in the early days [7]. Investors were hesitant to invest heavily in this field because they believed that the augmented world was yet to be adequately developed to yield the desired outputs [8]. However, many industries are gradually recognizing the need to invest in AR to remain at the top of the ladder and expand their brand, by attracting more customers with something new and innovative, as mentioned in ref. [9]. Since the introductory stage of AR, gaming has been its primary application. However, according to the report drafted by Goldman Sachs in 2016, AR is expected to improve retail, healthcare, and real estate markets in the coming years [10]. AR is used by various industries for product design; according to ref. [11], immersive service prototyping is in significant demand in the service design sector. AR has also been used in academics [12], aeronautics [13], and military [14]. It has a substantial potential to make every aspect of living enjoyable, easier, and more creative [15].

AR technologies are broadly classified into hardware, mainly consisting of varied displays and sensors, and the software algorithms required for integrating the augmentations with the real world. These technologies are used in several fields such as tourism and hospitality [16], education, medicine, retail, and gaming and entertainment. Hardware and software were integrated in the field of AR-based prototyping methods [17]. Integration is accomplished by accurately mapping a functional hardware prototype onto a virtual display. AR displays include optical projection systems, monitors, handheld devices, head mounted display (HMD) or head-up display (HUD), and eye tap. A handheld AR system was created to track optical markers in real time [18]. An optical projection system was generated via a mouse [19], enabling the configuration of input devices along with AR displays. HMD displays are described in ref. [20] as real-time three-dimensional interactive displays that allow free head motion and full body mobility; according to ref. [21], they are used widely as modelers. A usage method for HUD was provided by incorporating it into a laminated windshield [22], and it was patented. Spatial AR, the branch of AR that does not require displays to function, was studied in detail in ref. [23]. The authors of that study provided examples such as shader lamps, iLamps and mobile projectors, Being There, HoloStations, and smart projectors.

This paper presents a review of the use of AR in three applications: gaming, medicine, and retail. Gaming has been the leading sector in the use of AR, as a result of which gamers have experienced immense creativity, innovation, and unforgettable experiences. Gamers find AR-enhanced games better and more thrilling because of the engaging experience provided by the technology.

The use of AR in the medical industry has grown over the years. It has proven to be helpful to both doctors and patients. Patients can be educated about their diseases through AR, and the technology can also be used for complex surgeries, helping doctors to perform them with high accuracy. AR has also been used in the retail industry, and several companies have started investing in AR to create apps and amazing experiences to promote and sell their products. In-store technology, as well as online AR technology, has changed the way people shop. Different sectors of fashion that have been affected by AR and experienced retail change are discussed in this paper. AR has impacted our lives in previously unimaginable ways. Thus, it could be said that AR is the future of gaming, retail, and medicine. The expansion of the AR technology in these three sectors and its acceptance by the public was analyzed in this study. Surveys were performed and feedback from various customers was scrutinized to understand their perception of the new technology.

## AR in entertainment

The future of entertainment is likely to be influenced by advanced technologies such as AR [24, 25]. Mobile technological devices have made it possible for the entertainment industry to change the way people interact and engage with games, sports, tours, performances, among other activities. AR combines real and virtual worlds in 3D while being interactive [26–28].

In addition to redefining traditional gaming, AR is also already being used to increase the effectiveness of multimedia presentations and videos. However, it can be extended to a considerably greater array of entertainment fields, such as the way we listen to music and the way that we travel. Interface and visualization technology, along with some basic enabling technologies, are being incorporated to achieve heterogeneous and tangible interfaces [29]. AR may also be used collaboratively to display personalized information to each user. Further, it enhances broadcasting in sports events, concerts, and other events by highlighting or inserting information.

Ivan Sutherland was the creator of the first complete AR system with simple graphics [30] and a very heavy HMD. Subsequently, AR use in the entertainment industry has made tremendous advances, considering the latest well-known hit, which is an example of location-based gaming. AR completely changes users’ interaction, encouraging people to walk outside and read more, by transforming their books into an AR play space, whereas non-AR experiences limit users to a screen.

**Fig. 1:**
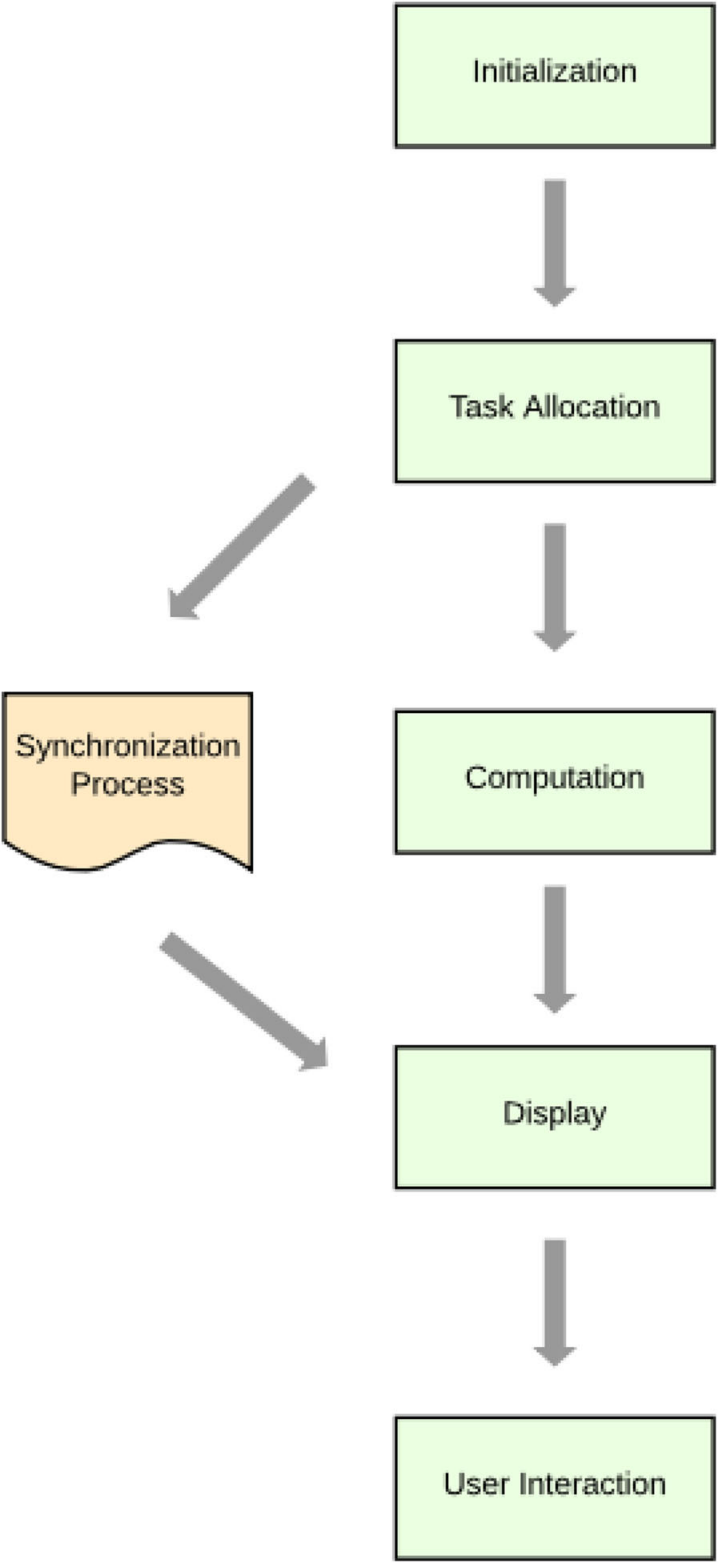
Data and workflow for mobile AR

**Fig. 2:**
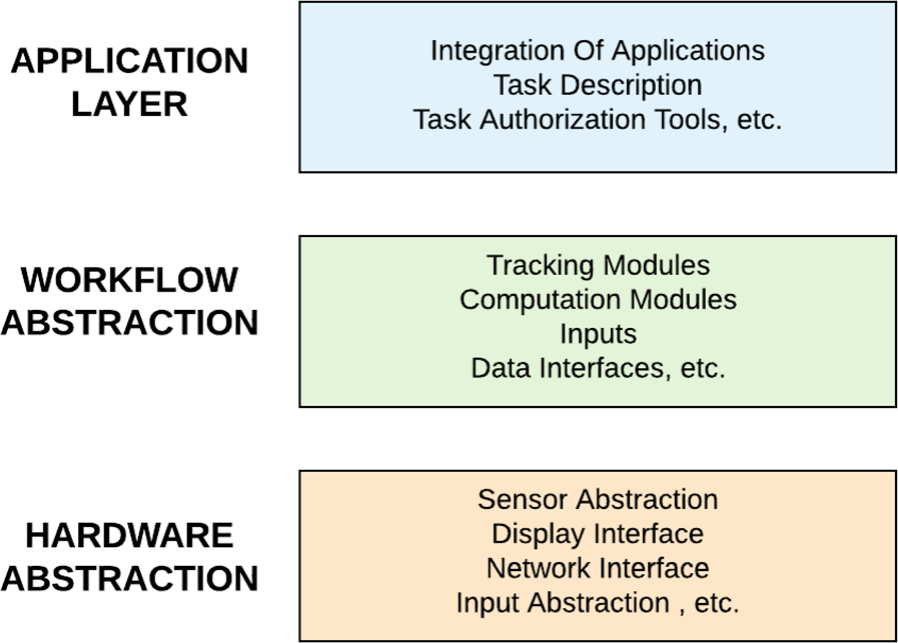
Framework for AR in mobile games

Most AR entertainment systems have software components that run on the device such as local game control and user tracking; server connection, which is often necessary in cases where there are shared resources, location-driven games, and where constant synchronization is required, is also used [31]. Although every system has its unique architecture, real-time performance can be achieved using cloud [19]. This data and workflow flowchart is depicted in Fig. 1, shown specifically for AR mobile systems. As shown in Fig. 2, every architecture mainly comprises three parts: layers that allow the integration of diverse hardware, application container, which is also a run-time context that contains application logic, including things such as navigation and assembly, and workflow abstraction layer, which is where all of the computational tasks occur, whether on the device or on cloud. The results of all these tasks are integrated with real contents and presented on the displays, which users interact with.

There are mainly two types of AR systems used for the purpose of entertainment. The first is marker-based applications, which are based on image recognition. This technology uses black and white markers that are used to detect the augmented object. To illustrate, the camera in a phone is first pointed at any marker’s position; after the marker has been identified, the required or embedded digital content is superimposed on the marker on top of the real object. Here, the images are to be coded into the system beforehand, making them easier to detect. Most AR apps seen in the market are marker-based. One of the most popular marker-based applications is Snapchat, which has attracted almost all of the population, and is very popular among the youth.

**Fig. 3:**
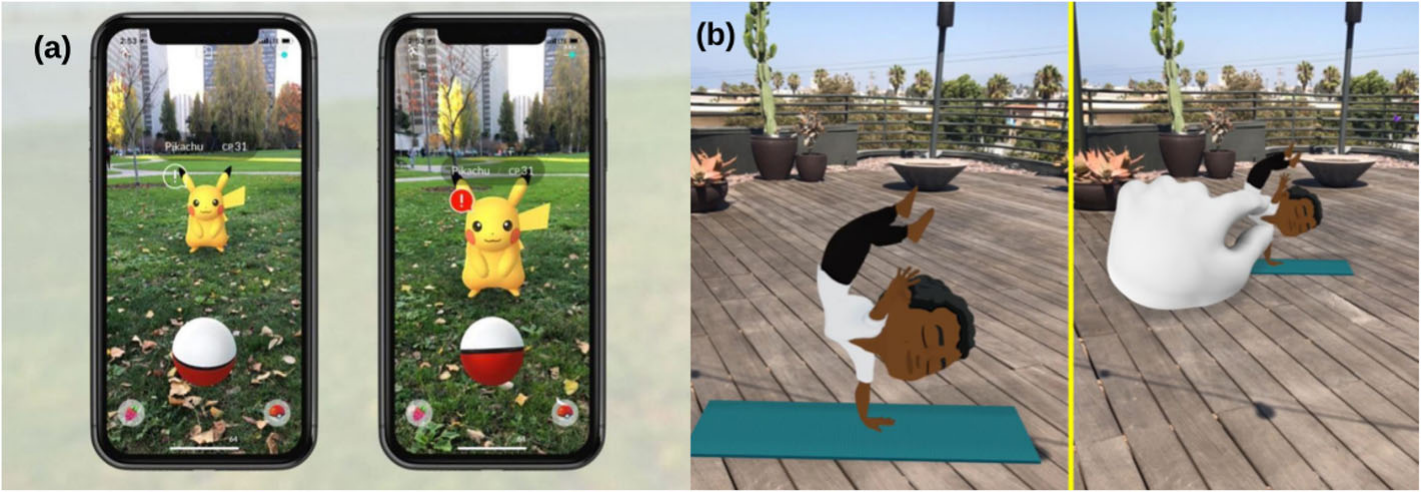
**a** Location-based game: Pokémon Go (Source: Forbes.com); **b** Marker-based application: Snapchat (Source: Vox.com)

The second is location-based applications, which work without markers. The technology makes use of global positioning system (GPS) or some digital compass that helps in detecting the user’s position, following which the real-world physical objects are replaced with or incorporated with the augmented objects. Such applications enable users to find the best restaurants nearby or locate their cars in parking lots. They can also be used in games that require the player’s location (Fig. 3).

**Fig. 4:**
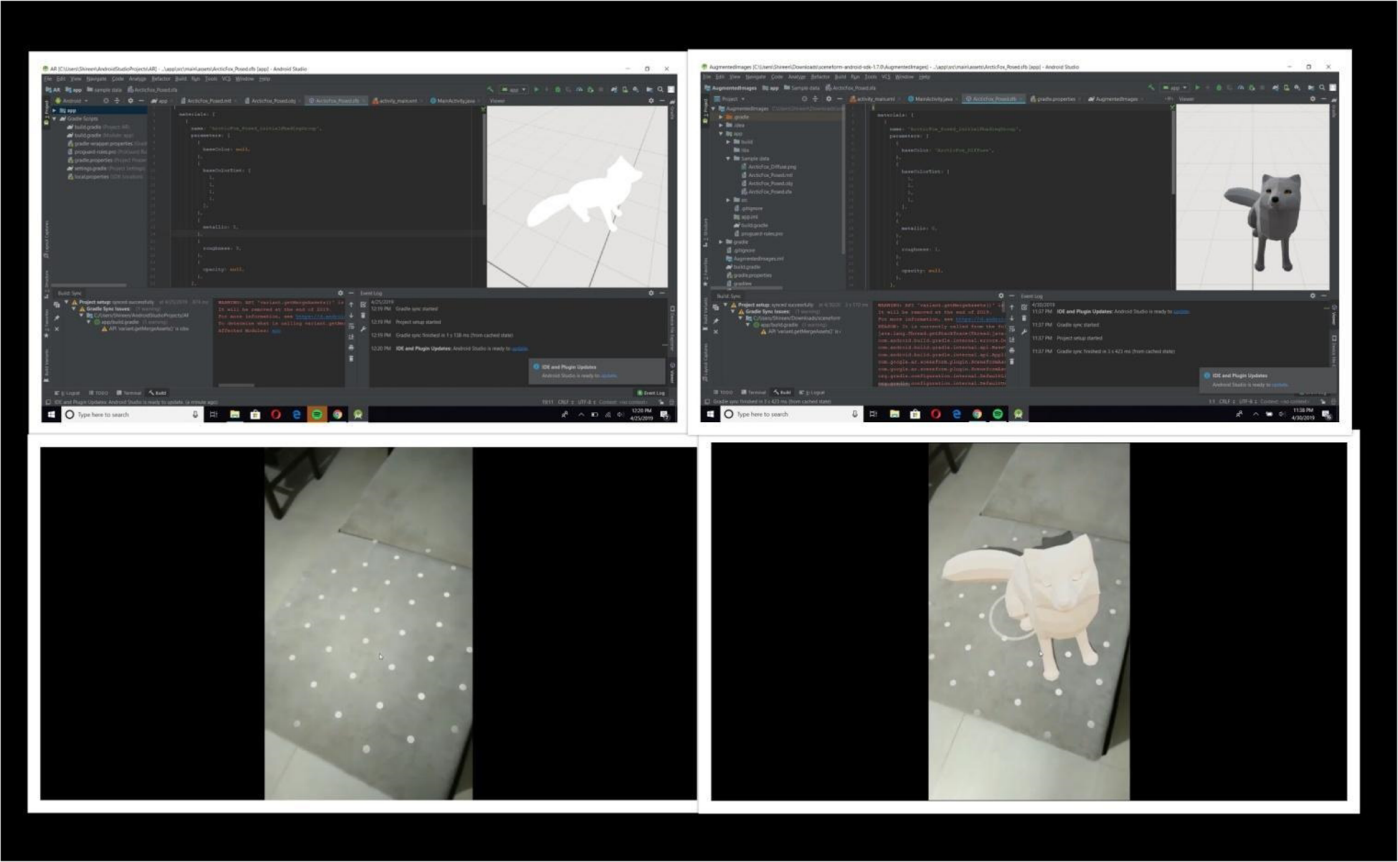
Implementation of simple application using Android studio, which allows users to project 3D animals into reality

Some popular software used to create AR applications are Unity, Vuforia, ARToolKit, Google ARCore, Android studio, and AR Spark studio. Unity is the most popular game engine used for developing games and AR apps. The aforementioned softwares are generally used by professionals and regular programmers. Figure 4 illustrates how a simple entertainment application that allows the user to project 3D animals into reality was implemented using Android studio.

The biggest application in entertainment is gaming. Although AR games may be limited in physical aspects and face-to-face communication, there is increased collaborative gaming and relationship building through remote multiplayer games [32]. There is significant potential for the emotional and mental aspects of a game, and AR makes it possible to create all types of scenarios and supports highly complex puzzles, models, and virtual opponents.

Despite the popularity of computer games, pervasive gaming, defined as gaming which increases physical movement and social interactions, enhances gamer experience [33]. This type of gaming focuses on the aspect of bringing virtual gaming back to the real world. One of the main goals of pervasive computing is to develop context-aware applications that analyze and collect information from the environment, as a result of which users alter their conduct accordingly [34]. This is achieved by using pervasive computing in combination with technologies, such as smart toys, and creating location-aware games that use the architecture that we currently live in as a game board.

**Fig. 5:**
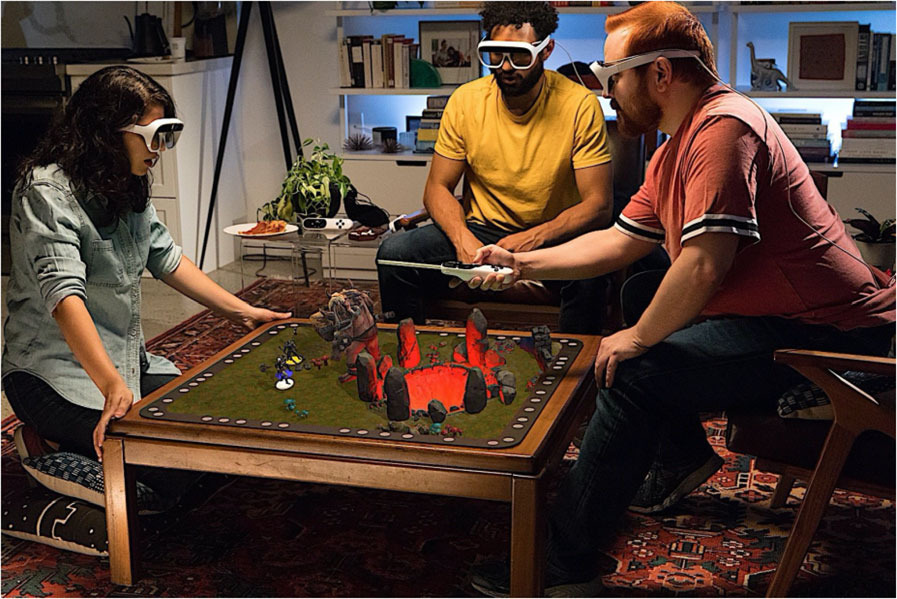
AR-based gaming

A local collaborative environment study, as shown in Fig. 5, in which multiple users could interact with the environment and communicate with each other at the same time using see-through HMDs and face-snapping, which allows fast and precise direct object manipulation, was conducted [35]. The gaming space was subdivided into spatial regions, and a layering concept was introduced for individual views and privacy management. It was observed that numerous board games and console games fit into this model, as a result of which they provide additional benefits and protect individualism and privacy.

Further, an emerging trend of serious games, which are computer games meant for non-leisure and educational purposes, has also been observed. They are different and better than traditional games because they may be used for simulations in areas, such as medicine, military operations, and education, thereby linking entertainment and work [36]. As case study, two AR games were used; the AR Puzzle, a puzzle game based on the City University campus in London, and AR Breakout, an old arcade game that was moved to a tangible environment. Based on the results collected, it could be said that compared to video games, AR games are easier to adapt to. It was observed that AR Puzzle turned out to be a very interesting and effective learning tool. In general, it was observed that tangible AR interactions were preferred by people over traditional ways of playing games.

AR games are also gaining momentum as learning guides, considering the younger generations’ immense use of media. They impact motivation and knowledge acquisition. As mentioned in ref. [37], real-world games, based on real and virtual elements, along with highly augmented computing functionality, create exciting and fun gaming experiences, potentially leading to high learning motivation. The ability of games to promote teamwork, collaboration, social interaction, and cooperation in a learning environment is frequently emphasized [38]. According to nine studies, the use of AR games in learning boosts learning performance and increases student motivation and enjoyment by 58% and 10%. However, the limitations of these systems, such as lack of interdisciplinary programs and students being distracted by the virtual novelty factors, persist [39]. AR-based learning has substantial potential, if proper approaches are developed based on study and analysis.

AR broadcasting is divided into two crucial elements: AR tracking and AR display. Although AR display techniques for broadcasting purposes are still nascent, they are used to project content into three-dimensional space. They are of three forms: head-mounted, monitor-based, and projection-based. Current AR tracking approaches are classified into three types: model-based, marker-based, and tracking without prior knowledge. Technologies, such as cameras, infrared sensors, hybrid sensors, and 2D and 3D markers, can all be implemented to identify a pattern and track its position in the real world. Robotic cameraman systems have been proposed to increase the quality of broadcasting systems and replace human operators [40]. It has been shown that robotic cameramen facilitate more precise and advanced interaction with virtual elements, and through zooming and multiple angle views, improve the performance of AR broadcasting in all sorts of environments.

An enhanced AR system displays statistical players’ information on captured images of a sports game [41]. It is an image enhancement technique based on an algorithm that implements multi-scale retinex. It was designed to improve the accuracy of player detection during adverse conditions such as intense sunlight. This is followed by face detection performed using adaptive boosting and Haar features for feature extraction and classification. Discriminant analysis and the nearest-neighbor classifier are used for classification. The system can also display player statistics. This model was tested on several images in immensely diverse conditions, and it was concluded that it could be extended to all types of sports where the inputs are images and the desired output is information displayed around recognized players.

A haptically enhanced broadcasting system that uses AR techniques to synthesize videos in real time, a multimedia streaming technology, and haptics were also implemented. The system operation sequence has four different stages: scene capture, haptic editing, data transmission, and display with haptic interaction. It can be used for creating haptic effects for cartoons and in the context of live sports broadcasts. The most noticeable feature of haptics is the sense of being social presence at the location displayed remotely. In live broadcasting, haptic interaction can enable an audience to take part in communication and discussions with those viewing the same program. Haptic interaction refers to the technology that creates an experience of touch through vibrations, motions, and the application of forces [42].

AR makes spectator sports more entertaining because of the additional information provided to the viewers. An AR-based sports system involves two major steps: homography estimation and automatic player detection, as described in ref. [43]. A marker-based approach that uses image patterns was designed for homography estimation, and a markerless approach that works for natural images with distinctive local patterns was designed for automatic player detection. For baseball fields, contours must be extracted and geometric primitives, estimated. For the player detection methods, an algorithm based on Adaboost learning, which is both fast and robust, was used. However, it failed to detect players sometimes. This system, which is based on still images captured using mobile phones, was implemented on mobile platforms. It made it possible to accept all the images taken from different angles, with large variations in the size and pose of the players, and different lighting conditions in the playground. Photos were taken with Apple iPhone 3GS, and a PC with an Intel 2.67 GHz Core I7 CPU was used to test the algorithm. In addition, Table 1 also discusses AR games, their advantages, and what technology was used to make them.

**Table 1:** AR in gaming

Title	Description	Results	Advantages	Reference
Tangible AR for computer games	Tangible AR provides a highly effective environment for multiplayer computer games. The communication between man and machine is simplified by the tangible user interface. The technologies used include the Studierstube and ARToolKit.	N/A	These types of systems combine the appealing animations and easy interactions of computer games and real-world board games.	[44]
Handheld AR games	The device’s movement and orientation were optically captured, relative to a visual marker; this enabled interactive control of mobile games in 3D. Recognition of multiple game-specific gestures was performed, and the mobile device camera view was augmented by overlaying graphics.	Handheld AR system architecture and game prototypes with specific hardware and technologies were described.	Handheld AR games are much more feasible, compared to HMD and other equipment, in terms of bulkiness and mobility.	[18, 45]
Audio AR system	Using an audio-only infrastructure, a game where the players can move in the real world and trigger actions in the virtual world was implemented. A simple wearable computer, along with an RF-based system, was used to detect the location of the players. Digital sounds corresponding to the current state of the user were played.	A prototype for a role-playing game that utilized a low-cost, lightweight audio augmented infrastructure was created. This infrastructure may be extended to other applications such as audio tours.	This system showcases immersive gaming environments, which can provide better experiences to gamers. Furthermore, it is effective for complex systems that require user interaction.	[46]
Multimodal AR tangible gaming	A tangible AR gaming environment that uses a multimodal tracking interface was presented. It was possible for the user to interact efficiently with the superimposed environment. It was also possible to implement six types of interactions: pinch glove interaction, hand position and orientation, head orientation, UMPC I/O manipulation and Wii interaction.	Two tabletop games based on AR were designed and implemented.	It enhances the gaming experience. Tangible games appear to be significantly more enjoyable than keyboard games.	[36]
Pervasive AR games to experience tourist destinations	The ExCORA mobile experience aimed to facilitate the engagement of the general public with the Urgull Mountain Spain. The aim was to encourage people to visit the mountain and to educate the visitors.	The game was tested, and it was concluded that the players can be immersed in the game, which had a simple interface.	Pervasive AR games provide an engaging and fulfilling tourism experience.	[47]
The MATRIS project	A real-time system that measures the movement of a camera was implemented through the MATRIS Project. The features in the scene from an inertial sensor were tracked by image analysis. The MATRIS approach was based on the way humans use the vestibular organs and eyes, which act like the camera.	N/A	No special infrastructure required for real-time camera tracking. It can be used in broadcasting and other AR applications.	[48]
Real-time AR system for sports broadcast video enhancement	Visuals for court-net sports, which were broadcasted on TV, were enhanced using an AR system. An expectation- maximization procedure was utilized to find the optimal feature points and acquire camera parameters from the TV image. A virtual camera was derived and mapping from the original camera was performed, making it possible to synthesize scenes from the players’ viewpoint,	The system was tested on six TV clips of sports such as tennis, volleyball, and badminton. The results obtained were promising.	The augmented content enables users to enjoy and comprehend the sports game better. The user can choose the viewpoint and engage better in the sports matches.	[49]
Enhanced broadcasting using AR in MPEG-4	Personalized immersive sports TV experience system aims at creating visual enhancements through AR and their embedding in the sports events footage in real-time or replay. This may be possible using an infrastructure based on MPEG-4, video processing, and computer vision techniques. The enhancements are then delivered over digital video broadcasting infrastructure.	N/A	Digitally interactive television offers new possibilities to viewers at home. The viewers may acquire a deeper understanding of sports events	[50]
Architecture of augmented broadcasting service for next-generation smart TV	Augmented broadcasting service for multimedia consumption, social network participation, and viewer-centric broadcasting. Viewers can watch the original broadcast and mixed broadcast, as per demand.	The architecture for this system was proposed in this study. This service has already been used in the virtual advertisement of many sports broadcasts.	Viewer-centric broadcasting is also possible.	[51]

## AR in medicine

As mentioned previously, the use of AR to enhance natural environments and alter the perceptions of reality is being exploited in various fields such as entertainment, education, retail, and marketing [52–54]. It is also being applied to the field of medicine. AR has been defined as a real-time indirect or direct view of the surrounding world that has been augmented with computer-generated virtual information [55]. AR is indeed highly beneficial to the medical field; however, considerable effort and care must be taken to reap its benefits. The use and function of AR in medicine depends on the skill of the technician, as well as that of the doctors and medical teachers involved. AR systems are also extremely costly, compared to the normal medical methodologies. Hence, to reap maximum benefits, the AR systems must be deployed with significant care and accuracy [56, 57].

Ref. [58] discusses the importance of AR and VR in the fields of medical anatomy and health sciences. The purpose of this research was to assess whether medical students who used VR and AR were more effective than those that used other mobile applications. Fifty-nine participants were randomly assigned three learning modes: VR, tablet-based applications, and AR. The senses of the users using VR are fully immersed in a virtual environment that mimics the properties of the real world through HMDs, stereo headphones, high-resolution motion tracking systems. AR, on the other hand, is used to superimpose digital models on the real world. 3D tablet displays are used mainly for user interaction. Using these teaching modes, a lesson on skull anatomy was conducted. The anatomical knowledge of the medical students was assessed through a repetition of experiments with different lessons. It was noted that both AR and VR were more beneficial, as they promoted increased engagement of the medical students.

On the other hand, ref. [59] conducted a review that evaluated the past, present, and future of the usage of computer-aided AR in surgeries. Computer-aided AR, also known as computer-aided drawing is a drawing tool that allows the user to make accurate data models using AR. The review centered on the different types of surgeries where AR can be used as a display or a model. A systematic review of the effectiveness of AR applications in medical training yields a promising outlook as well [60]. The training applications were assigned to three different categories: echocardiography training, laparoscopic surgery, and AR and VR training for neurosurgical procedures. This literature suggests that although AR may have gained scientific interest, no recorded evidence suggests that AR can transfer information to the user seamlessly and promisingly.

**Fig. 6:**
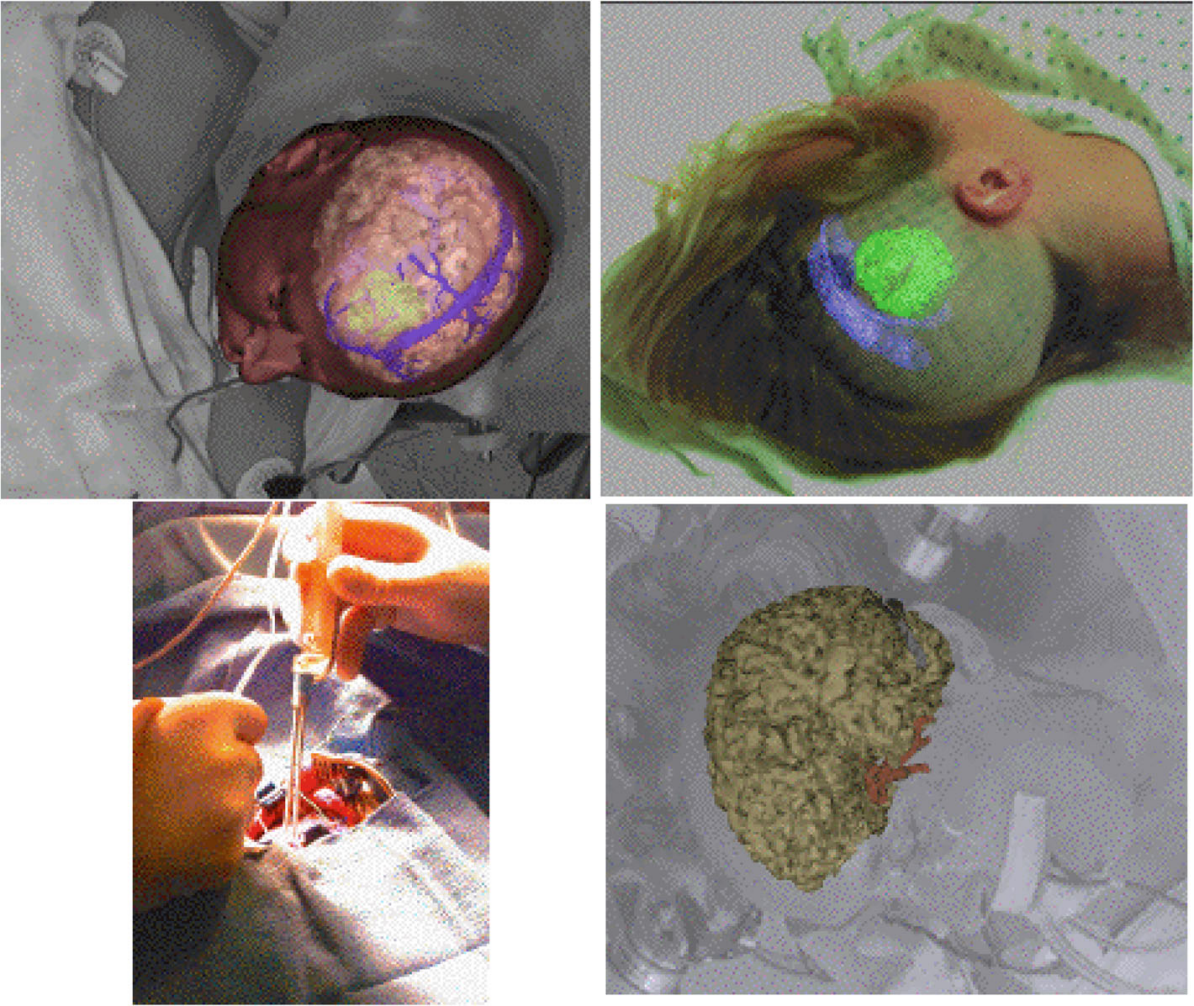
Brain imaging and brain surgery using AR

Medical displays and accurate medical imaging technology are significant because they enable physicians to fully exploit rich sources of heterogeneous intraoperative and preoperative data (as shown in Fig. 6 which depicts intraoperative brain imaging system). Ref. [61] discussed these advanced medical displays and also established a relation between the subsets of such bodies of work, to give an idea of the challenges that may occur during the application of such displays. They discussed AR technologies, such as HMD-based AR systems, augmented optics, augmented windows, monitors, and endoscopes, and their specific applications in the medical field. In the study, the solutions that can be provided by AR were acknowledged, and its use in the workflow was encouraged. HMD-based AR headsets consist of OLED microdisplays on which AR systems, such as augmented optics and windows, can run.

Surgeons are often the earliest adopters of technical tools that can enhance the surgical and patient experience. The application and limitations of a digital surgical environment that uses AR and VR were discussed by ref. [62]. The applications include operative benefits, broadcasting and recording of surgery, anatomical evaluation, telementoring, and provision of medical education. Limited battery life, large devices, and cumbersome cables are the limitations of the technology. However, it has been stated that significant progress will be made in the coming generations with the development of these tools, which may potentially lead to an increase in their usage as surgical loupes.

An ophthalmic AR environment was developed to allow for more accurate laser treatment for ophthalmic diseases, telemedicine, and real-time image analysis, measurement, and comparison [63]. The system was designed around a standard slit lamp biomicroscope. A camera, interfaced with the biomicroscope, was used to capture the images, which were then sent to a video capture board. The image was processed using a single computer workstation, and fast registration algorithms were applied to it. The output given by the computer was a VGA resolution video display with adjustable contrast and brightness attached to the oculars of the slit lamp microscope.

A medical AR system performs three tasks: camera or instrument tracking, patient registration, and creation of preoperative planning data. A video see-through system for medical AR was described in ref. [64]. The system was based on a VectorVision image-guided surgery device. They demonstrated that their system could perform all the above mentioned tasks. VectorVision is an optical tracking IGS platform consisting of two infrared cameras, a PC, and a touch screen display. A vector vision link is a TCP/IP-based interface that is integrated and used with the vector vision cranial system. The tests showed that an augmented video stream with an average frame rate of 10 fps was generated by the augmented video stream using a 640 × 480 pixel webcam. Furthermore, they recorded a latency period of approximately 80 ms, and the camera tracking method exhibited good accuracy. Hence, they provided a novel approach for realizing AR applications in the medical field.

A specific technology that is used extensively for visualization is the HMD. Ref. [65] discussed AR visualization performed through the use of a head-mounted operating binocular required in the field of medicine. The head-mounted operating binocular is a somewhat modified version of the HMD. The radioscope was adopted because it is a miniature and cost-effective system that can be conveniently deployed for visualization. In this study, a basic design of the modified HMD was displayed, and the results of a detailed laboratory study for photogrammetric calibration of the varioscope’s computer display to a real-world scene, was presented. The location of a position measurement probe of an optical tracking system was transformed to the binocular display with an error of less than 1 mm in the real world in 56% of all cases. In other cases, the error was found to be less than 2 mm. Hence, we can conclude that sufficient accuracy was achieved, such that it could be applied for a wide range of CAS applications.

A haptic AR environment was used to design cranial implants, as described in ref. [66]. A haptic AR environment conveys the sense of touch to the user; ‘haptic’, in general, refers to any technology that provides the experience of touch through motions, vibrations, and forces. The data obtained from the patient CT was used to create virtual 3D cranial models that were superimposed over their hands. Through such an environment, the medical cranial sculptor could feel and view the model. The personal augmented reality immersive system (PARIS), a new prototype display system, was also used alongside the models. The PARIS system creates the illusion of a 3D tool that can be held by the sculptor. Neurosurgeons, paleontologists, and radiologists have expressed interest in utilizing the system.

**Fig. 7:**
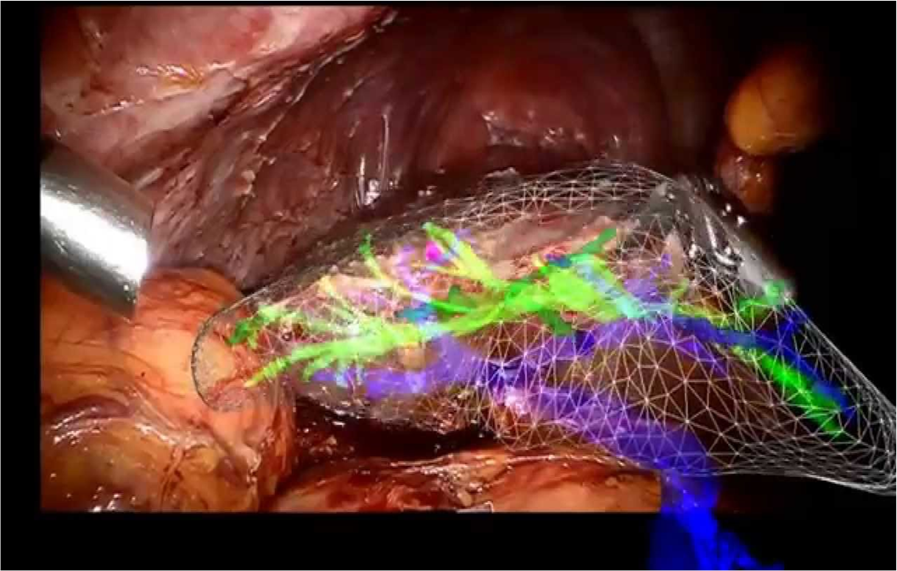
Hepatic surgery using AR

Ref. [67] (Fig. 7) presented a paper on an AR system for thermal ablation of the liver. This system was first evaluated on an abdominal phantom, and subsequently, on patients in an operating room. The preoperative image of patients and the needle position that a medical practitioner manipulates were registered in a common coordinate system. The feature points were extracted and processed through validated algorithms. The experiment showed that a predefined target with an accuracy of 2 mm could be achieved at an insertion time of less than a minute. The output inspired confidence that the system provided accurate guidance and information during the complete breathing cycle of the patient.

A rehabilitation system for hand and arm movement was implemented through a spatial AR system, as described in ref. [68]. The system created a virtual audio and visual experience by tracking a subject’s hand during rehabilitation-related tasks that involve the elbow, shoulder, and wrist movements. Real-time data and photos were sent to the clinic for further evaluation and assessment of the system. The study proved that the system was functional through the application of the technology in the laboratory. The system made it possible to incorporate real objects into tasks, as desired. It also controlled for external objects and ensured the safety and comfort of the patients. Another advantage provided by the system was that the tasks could be modified by a therapist based on the needs of the patient. The system they outlined depicted a performance-driven exercise program for stroke rehabilitation.

Apart from medical teaching and anatomy, medical surgery is an essential use case of AR in medicine as well. Table 2 shows such essential examples of AR in surgery.

**Table 2:** AR in surgery

Title/ operation performed	Human organ/ type of surgery	AR technology used/ description	Conclusions/results	Reference
Image-guided navigation through an AR system	Oral and maxillofacial surgery	Different types of projection-based systems, such as SMN, and screen-based systems (VectorVision) were discussed.	Two operational cases where AR has been successfully used were discussed.	[69]
3D stereoscopic visualization using an AR system	Oral Surgery	A 3D integral video (IV) display system and an IV AR System.	An 80-fps IV image was rendered with minimal error. Screw fixation was also effectively performed using the AR system	[70]
An intraoperative brain imaging system used for neurovascular surgery as shown in Fig. 6.	Neurovascular surgery, specifically, aneurysm surgery, arteriovenous malformation, arteriovenous fistulas	A workstation, an optical tracking system such as IGS and camera	Patient-to-image registration error was approximately 3.44 mm; calibration and rejection error was 2.02 mm. Overall AR misalignment was found to be 1–2 mm.	[71]
Computer-aided hepatic surgery planned using AR. This is performed on the liver as shown in Fig. 7.	Liver surgery and operation of liver tumors	Stereoscopic see- through HMDs, tracking systems, a rendering and tracking workstation	A table providing the interactive volumetric measurements of the liver vessels and tumor was generated. The outlined AR tool can be considered an excellent tool for surgery planning.	[72]
AR use for navigation in computer-assisted arthroscopic surgery	Post-surgical recovery of the knee	Consists of two operating modules. The first module consists of preoperative image processing, and the second is used intraoperatively.	The system was tested using an artificial model of a human knee. The virtual model obtained following segmentation using Module 1 and 3D reconstruction using Module 2 had minimal errors. Although further testing is required to validate the system, the first trials yielded positive feedback.	[73]
Natural Orifice Transluminal Endoscopic Surgery (NOTES)	Performance of surgical management of disease in the abdomen	The image registration technique was used along with NOTES. Through this, we could guide and position the probe in the desired orientation.	A series of experiments reveal that the display of a spatially matched reformatted reference image and the presentation of probe position in the 3D models provide valuable support to the operator in the navigation and positioning of the probes.	[74]
VR and AR in digestive surgery	Detection of parenchyma and tumors, digestive surgery	A multimedia computer was used to model a surgical planning system in 3D. CT scan and MRI scan data were run on the system, and the outputs were modified.	The overall accuracy of the system was found to be less than 5 mm. Hence, the results of the experiment clearly showed important targeting accuracy. The use of the system required an average time of 30 s, compared to the clinical procedure, which takes 5–10 min. Hence, a fully efficient and accurate image-guided surgical tool was obtained.	[75]
Assessments and considerations of the use of AR in cerebral surgery	Cerebral arteriovenous malformations (AVM) surgery and performance of AVM resections	Angio-CT and angio-MRI were implemented over an AR Iplan platform that uses BrainLAB	The following system was operated in five cases where different resections were required. Postoperative and preoperative ranking orders were generated as well. All the resections were successful, except in the case of one patient who was pregnant during her AVM operation.	[76]

## AR in retail

AR significantly impacts how companies compete with one other in the technologically advancing environment. AR, as a result of its growing acceptance rate over the years, has heavily influenced brand awareness and expansion. The concept of AR in retail is anything but new. According to ref. [77], some of the largest firms, such as Coca-Cola, McDonald’s, and General Electric, have invested in AR for better retail experience and more innovative ways of marketing their products. The sales department of Coca-Cola collaborated with Augment to deploy AR to build a software that could help visualize the look of coolers in retail stores. This will help B2B customers make better product choices. Trigger developed an AR app for McDonald’s by bringing selected few animated figures and characters to life for an interactive experience for children. The software platform used was Vuforia. The main aim of the app was to feature characters from DreamWorks movies, such as How to Train Your Dragon and Mr. Peabody and Sherman, on an AR platform to make kids experience healthy fun. The surface around the Happy Meal box would come to life, and a garden filled with cherries, apples, tomatoes, and carrots would emerge.

The best way to compete healthily in the market is to build strong relations with customers and gain their loyalty by enhancing their engagement with the products. Ref. [77] talks about three different types of consumer engagement facilitated by AR. User-brand engagement occurs between a customer and the product that he/she wishes to buy. This type of engagement could be made as immersive as possible, allowing the users to manipulate and interact with the technology. User-user engagement helps customers interact with each other based on the AR content. They can modify each other’s digital data, resulting in the strengthening of their bond, as well as their individual relation with the company. User-bystander engagement enables customers to make artifacts of their experience with AR and share them on a social platform, thus leading to the advertisement of the product, which in turn benefits the company.

**Fig. 8:**
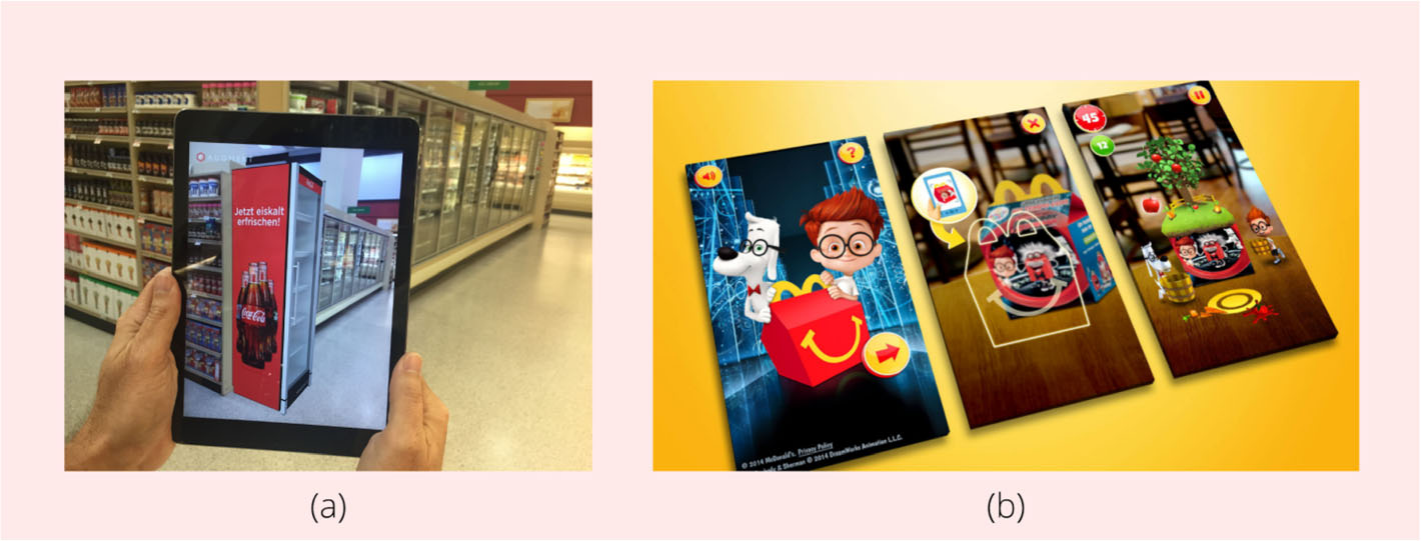
**a** AR Use by Coca-Cola (source: augment.com); **b** AR Applied to McDonald’s Happy Meal box (source: triggerglobal.com)

As mentioned in ref. [78], AR has expanded into various forms such as HMDs, mobile applications, contact lenses, and devices. One such smart device is the Memory Mirror, set up by Neiman Markus, which helps customers look at outfits from different angles and compare the various selected outfits simultaneously (Fig. 8).

It has been demonstrated that AR can help build customer relations and boost sales by reducing the risks that customers face while purchasing a product. Ref. [79] discusses how AR can improve customer insights, make the shopping experience enjoyable, and reduce the customer perceived risks. The perceived risk is the uncertainty faced by the customers or the negative results they might get from the purchase of a product. A research model indicating that AR can indeed help in reducing the various risks was proposed. The entire model consisted of six different dimensions of risks to be eliminated or reduced through the use of AR. These include social, financial, psychological, performance, physical, and time risks. Thus, it has been assumed in this paper that AR can reduce the perceived risks but empirical proof is yet to be given.

**Fig. 9:**
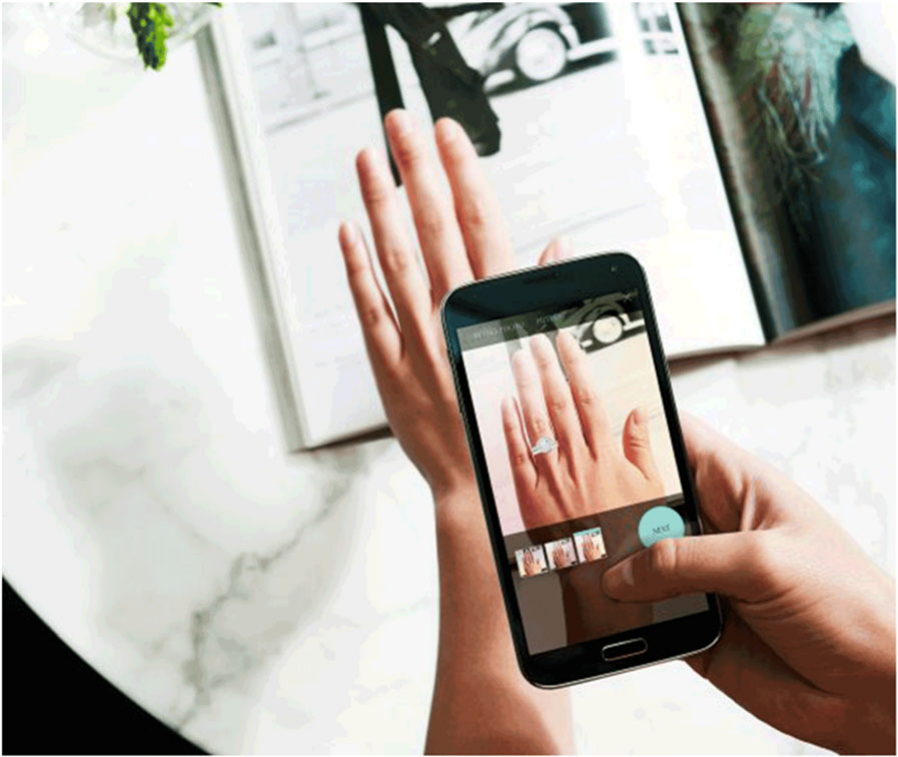
AR-based mobile app for online shopping

According to ref. [80], retailers have lost sales to online shopping over the years. However, with the introduction of AR, retailers can reinvent the customer experience and make it far more interesting than traditional shopping. Furthermore, this study also centered on price optimization. Loyalty programs help retailers keep track of customer identification and provide the customers with discounts in return for their data. Thus, integrating AR with loyalty program data could help retailers optimize the prices of products, according to a specific customer. Such personalized shopping experiences could improve the customer experience; further, such AR systems could help the customers navigate easily through the products that are affordable to them. Thus, with the ease brought on in the shopping experience, the customers might prefer going to stores to shopping online. However, an increase in use of AR in online shopping apps has been also gaining momentum for example a jewellery app, as shown in Fig. 9.

**Fig. 10:**
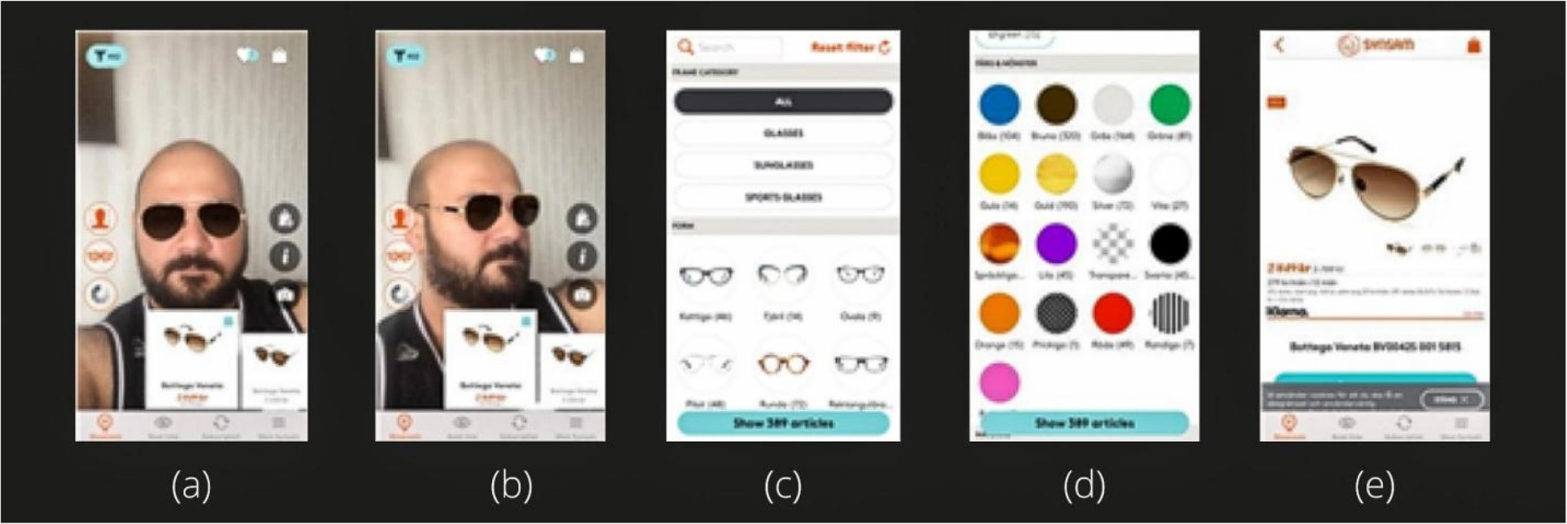
Virtually trying on glasses using synsam AR application

Using an app launched by a Swedish eye retailer, Synsam, as shown in Fig. 10 [81]. studied the impact of an AR-based smartphone application on the customer’s product purchase intentions. The app helped the customers try out different eyeglasses without actually putting them on physically. Their survey aimed to investigate whether the digital experience had a positive effect on the decision to purchase and the determinants that led to it. It was reported that many people found the experience very helpful and fun. It was observed that the females enjoyed trying out different pieces of eyewear using the selfie feature, whereas, the males were more fascinated by the technological side of AR. However, there were people who felt that going to a store and trying on the eyewears physically before buying them was better. However, a significant percentage agreed that AR was a useful technology for buying products.

**Fig. 11:**
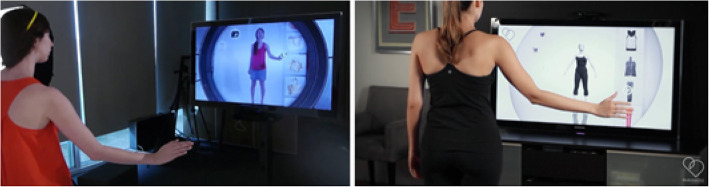
VFR (source: ref. [82])

The introduction of virtual fitting rooms (VFRs) has taken AR to new heights [82]. These VFRs enable a person to try on outfits without actually being present in the store. This concept can also be used for in-store shopping, making the customer experience fun and easy. A combination of a variety of technologies, such as natural interaction (NI), 3D scanning, 3D models, and omnipresent social networking features, have been used to make the idea of VFRs a considerable success. NI enables users to interact with the augmented environment using hand gestures, speech, and body language. 3D sensors are used to scan a user’s body to create a 3D avatar-type model, which is then integrated with other data, such as gender and different retailers. Customers can be granted access to a variety of clothing, creating a real-time shopping experience. As shopping can be-time consuming and exhausting, adopting such innovative ways can make the customer experience more interactive and fun and less tiring. Furthermore, the biggest obstacle to online shopping concerning whether the garment would fit or not, can be eliminated by such future VFRs. Figure 11 depicts the possible look of a VFR.

**Fig. 12:**
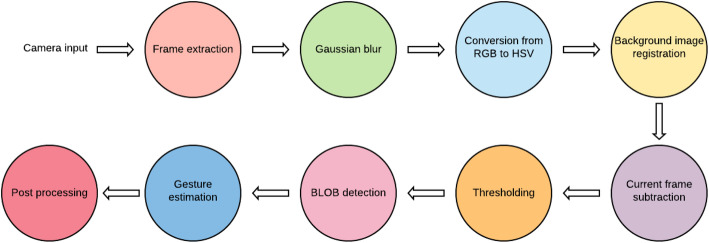
Working of virtual trial room based on steps mentioned in ref. [83]

Ref. [83] presented an AR-based virtual trial room that allowed the user to try on clothes virtually. This study aimed to enhance the online shopping experience and reduce the time spent on in-store shopping by decreasing the queuing time. In this method, a human was detected from the background using light variations. Frame extraction, blurring, Red, Green, Blue (RGB) to Hue, Saturation, value (HSV) conversion, current frame subtraction, thresholding, Binary Large Object detection, gesture estimation, and post-processing were the steps that made up the desired system (Fig. 12). Relevant frames and data were extracted from the camera input, following which the Gaussian blur was applied, to remove unnecessary image noise. This was followed by the RGB to HSV conversion, to achieve greater accuracy and image registration, where different sets of data were transformed into one coordinate system. Then, frame subtraction was performed to reduce the background noise and emphasize the foreground details. The gesture estimation step familiarized the system with general gesture functionalities such as “try next cloth”, or ‘dislike’, or ‘like’. The final post-processing step was necessary, to add final touches to the output. This model could be further enhanced by adding social features that enabled users to take pictures and share them with friends or family.

Ref. [84] discussed how AR had impacted the customer experience; with the aim of integrating psychological, technological, and behavioral perspectives, an embodiment-presence-interactivity cube was proposed based on a variety of existing technologies. AR has an important role to play at every stage of the customer experience. In the pre-experience stage, customers obtain detailed information on the products, which enhances their decision-making. In the experience stage, the shopping experience is made more immersive and enjoyable for the users. The post-experience stage enables customers to evaluate their experience, create content, and share with other people. This leads to customer loyalty and brand awareness.

The effects of AR on customer behavior in purchasing a fashion product have been mentioned in ref. [85]. An experiment involving 162 participants aged between 18 and 35 was conducted. They took the help of an AR app of a makeup retail brand, which enabled the participants to apply different makeup to their faces using a virtual mirror. The entire experiment session included interaction with the app, following which questions were posed to the customers on their experience and purchase intention. It was observed that people who had experienced augmentation shared positive feedbacks on their experience and purchase intention. The customers who were hedonically motivated experienced more positive emotional response. The present model can be extended to include more features and yield more outcomes beyond purchase intention such as customer satisfaction and loyalty.

Ref. [86] discussed how fashion retail has evolved and how the emergence and growth of technology is expanding the fashion retail market. In the past, retailers had to create large portfolios and have a big store space to gain the attention of the users. Further, they explained how the introduction of online shopping was a revolutionary step that changed the scenario of a shopping experience. At present, with the advancement and acceptance of AR, brands are including AR in their strategy for remaining at the top. They proceeded to discuss omni-channel retailing, which consists of a cross-channel customer experience, through which a user can access multiple retail channels and also use multiple devices. Ref. [87] also discussed the penetration of the fashion industry by AR, leading to its growth in technological aspects. Further, ref. [87] mentioned the acceptance scenario of the AR technologies. The technology acceptance model (TAM) was the first model to focus on the insights of why the customers may accept or reject a new technology. The TAM has been used several times for research purposes, as it is a very helpful model in determining user acceptance. Ref. [88] also reviewed the implementation of AR by retailers, its applications, and consumer acceptance. The TAM model has been used to determine user acceptance and highlight the need for efficient and consumer-friendly devices in the future for retail growth.

Table 3 discusses different AR applications and devices created for retail experience, and surveyed upon by their respective developers along with the kind of response the technology received.

**Table 3:** AR in retail

Survey topic	Description	Results	Reference
AR-enhanced fashion store (sportswear retail store located in UK selected as a case study)	Enable virtual access to the store virtually, product visualization, and welcome customers by engaging them with AR at the entrance	Customers found the experience very interactive, engaging and fun. It led to better brand engagement and customer satisfaction	[89]
Use of android-based AR mobile apps for a smart retail experience	Analyze how AR apps affect customer experience and benefit the retailers	Shopping experience was found to be more efficient and beneficial in various ways	[90]
IKEA AR application	To understand the AR marketing phenomenon, different determinants, such as purchase intention, attitude, hedonic value, telepresence, ease of use, and technology anxiety were calculated	The outcome was positive on the customer experience side. It was further observed that users preferred apps that were easier to use. Furthermore, it was noted that women shopped for pleasure, whereas men emphasized the purchasing decisions	[91]
AR application for a German bookseller	The users got detailed description of the book at which they pointed the app, along with book cover and other specifications	A few limitations were observed; for instance, the price-related variables could not be collected, as there was a fixed price norm in Germany. The participants felt that having a trained salesman would be helpful for people who did not know how to operate the new technology. On a positive note, it was noted that AR had a potential to improve the extraction of information at the point of sale	[92]
Virtual dressing room based on depth data	The project was implemented using Unity, a popular framework for 3D applications. Microsoft Kinect was used for the tracking processes, and OpenNI and NITE middleware were used for fundamental functions. Furthermore, different backgrounds for trying on clothes were made available	Overall, the participants found the concept of the virtual dressing room to be very efficient, as it allowed them to scan through multiple clothes in a short period of time. The downside of the experiment was that the users could not determine whether the virtual clothes would fit them in real life. Furthermore, there were some users who were not very comfortable with computer technology, and, hence, took time to interact with the device and get used to the entire set up.	[93]
The study used two conditions from the Ray Ban’s website and five conditions from the star mobile applications, which were all AR-based	Selected participants were randomly assigned to one of the AR groups. All of them were asked questions before their interaction with their respective app to get an idea of how aware and familiar they were with the technology. After their interaction, they were asked to describe their experience with the AR technology	The Ray Ban webcam model generated the highest level of satisfaction because of its high interactivity and AR features. The other model of the Ray Ban website used, on the other hand, earned the lowest level of satisfaction because of less interactive features. From the results obtained, the attributes that affected the consumer satisfaction were identified.	[94]
Dynamic Fitting Room using Microsoft Kinect and AR technologies	The users could select the clothes or interact with the devices using gestures. The user’s cloth size was automatically measured based on different brands and different country standards.	Before the experiment, the users were asked about their clothes size; they were then automatically measured by the augmented room. The value measured was very close to the actual size. The proposed system was found to be useful in reducing the shopping time	[95]

## Applying AR in fight against COVID-19 crisis

**Fig. 13:**
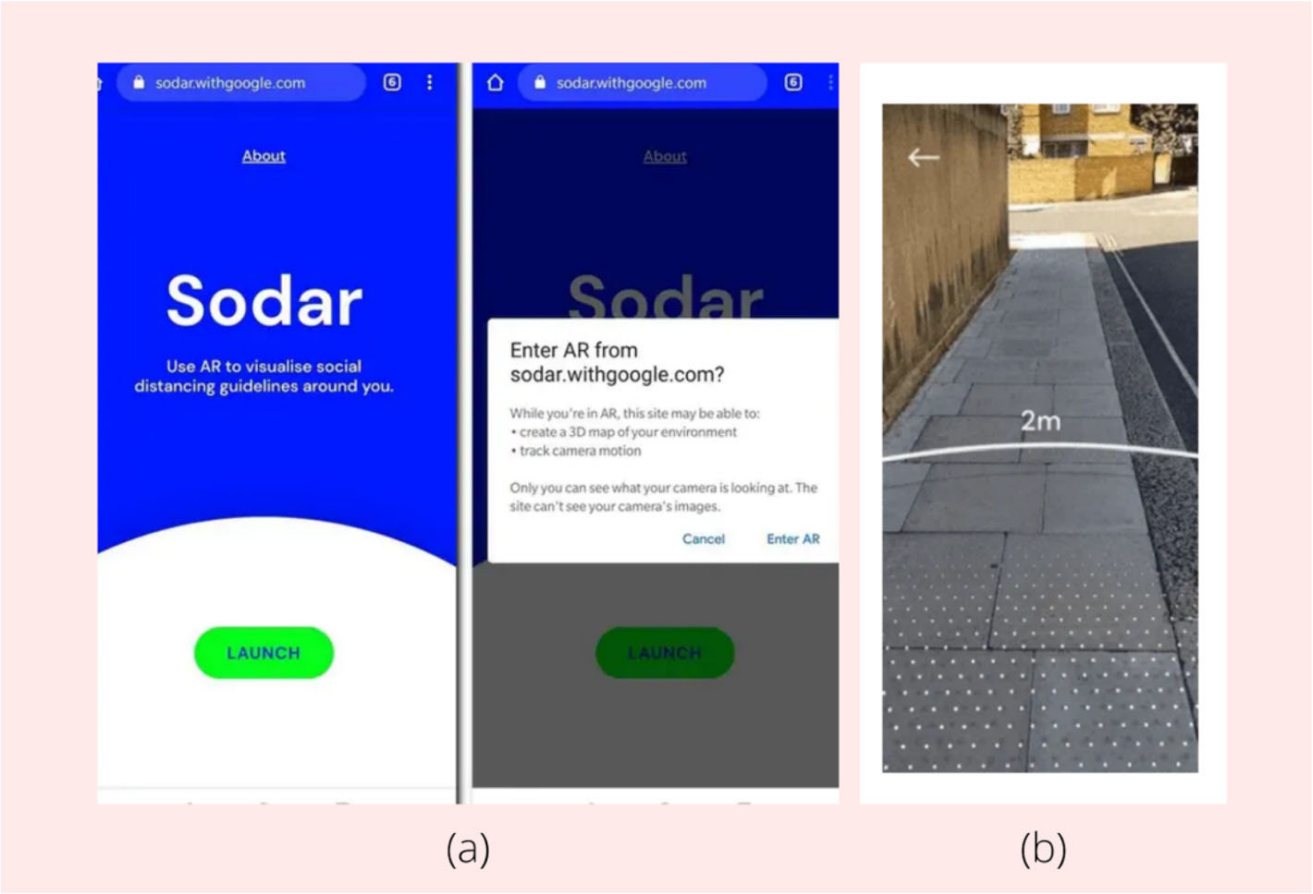
**a** Launch of sodar application; **b** 2-m radius being displayed after camera is pointed toward area

The COVID-19 virus has spread to the entire world. It has caused a significant number of deaths and significantly changed the lives of the people who have been affected by it. Many countries have gone into lockdown to prevent the spread of the virus, thereby resulting in economic collapse within them. Many businesses have shut down, and schools have been closed. A lot of measures have been taken to reduce the effects of the virus and, expectedly, the scientific community is developing various technological methods that can benefit the society, amidst these trying times. For example, a framework for change was proposed for medical education [96], and ref. [97] discussed the monitoring of hospitals and clinics through technological methods. AR can be very useful for navigating life during the crisis. Sodar, an AR application that upholds social distancing by helping individuals maintain a distance of 2 m from other people, was launched by Google (Fig. 13). Such an application will prove to be very useful once the lockdown ends and people start to go outside again.

**Fig. 14:**
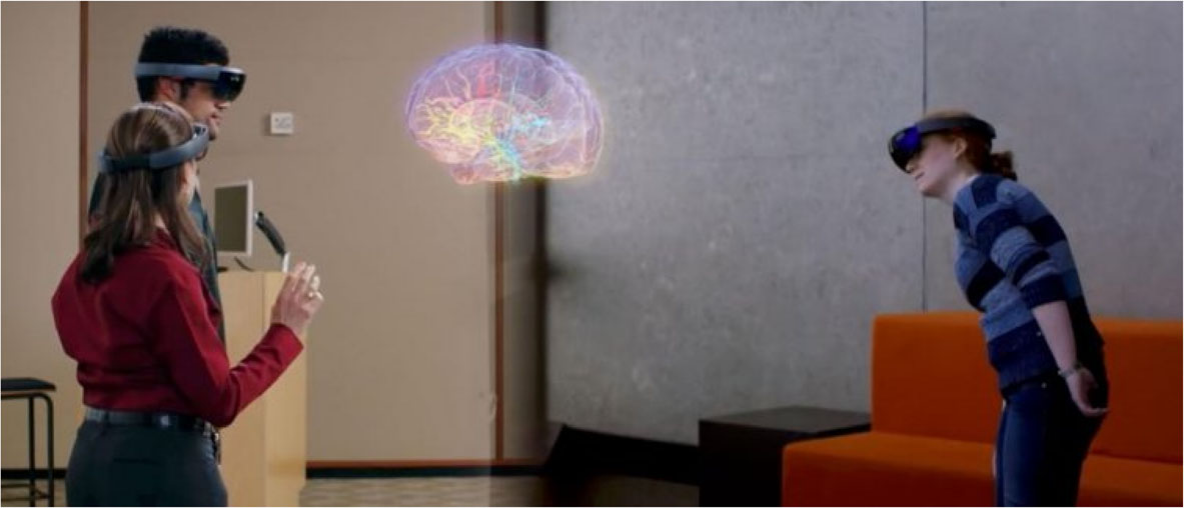
HoloAnatomy AR system (source: CWRU website)

Case Western Reserve University, in collaboration with Cleveland Clinic, developed an AR app called HoloAnatomy, which helps medical students to learn about the human body in 3D. HoloAnatomy teaches students anatomy using Microsoft Hololens (Fig. 14). The students can learn about the smallest details in the human body without having to dissect cadavers. Such online educational AR apps can be extremely useful in the current COVID-19 crisis.

AR can also be used to develop long-distance healthcare systems for managing the pain and wellbeing of patients suffering from chronic pain and health care issues due to the COVID-19 outbreak. Telemedicine and web-based systems are some existing prevalent approaches. Telemedicine refers to the short message services, video conferencing, and telephone consultation. Web-based systems, such as PAIN OUT in Europe and CHOIR in the United States [98], make it possible to review patients before appointments. However, they depend on customer inputs and lack functionality. AR involves a projection being mapped onto the physical world to improve perception and give acute vision to doctors to facilitate the speedy recovery of patients [99].

AR can also be beneficial in the surgical field, as well. Virtual technology is responsible for saving lives and safeguarding surgical practices during the pandemic; the Proxemie platform is one such example [100]. The Proxemie platform connects surgeons to a live environment through which experts can provide support to their colleagues and supervise procedures. Proxemie’s AR telehealth solution is used for conducting multidisciplinary meetings to assess patients. The platform also provides a surgical library that provides useful information on surgeries. Hence, it is an extremely essential platform and a suitable example of the usefulness of AR in the current pandemic.

**Fig. 15:**
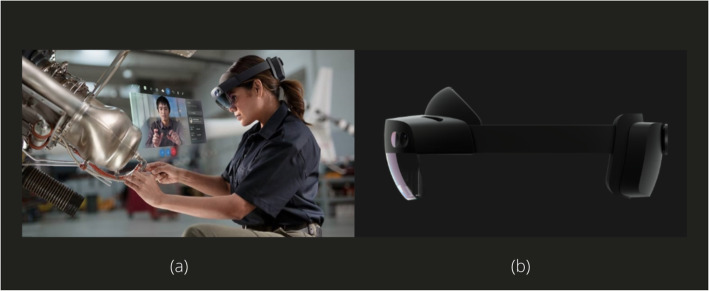
Dynamic 365 remote assist on Microsoft Hololens 2 AR headset

However, a more significant challenge awaits the society at the end of the pandemic. The road to recovery from the pandemic will be extremely difficult. AR software and hardware shall be used to mitigate such effects even after the pandemic is long over. AR can be used to impact practical knowledge because the processes of learning and implementation. Using an AR headset, a skilled technician can seamlessly guide fellow workers and teach students. Companies can also train their workforce using AR, thereby improving their workflow and the economy. For example, Microsoft Hololens 2 AR headset can be used by companies to guide their employees (Fig. 15). It provides hands-free visual assistance and data, along with robust security and collaboration with other Microsoft apps. Companies that depend on on-site technical maintenance for their cash flow need AR solutions as well. AR-assisted service prevents physical contact and encourages social distancing, thereby satisfying the requirements of the present and the near future.

Another important area where AR can prove necessary is in retail, from the customer’s perspective. Whether online or in-store, people will never buy products without being sure if a particular product fits them. AR-based technologies, such as VFRs and mobile apps, can enable the customers to try out clothes, jewelry, makeup, sunglasses, or shoes, without actually trying any of these products in reality. Such AR-based solutions will help people practice social distancing. Digital and safe shopping experiences are among the current needs of customers. As explained previously regarding various AR systems used in retail, such applications can prove to be really useful in the coming days, thus boosting the AR market.

## Challenges and future scope

Before AR can be accepted by everyone on a large scale, it is important to note that AR faces a large number of challenges that must be overcome for it to thrive. Every technology consists of a well-defined business model based on which investments are generated. However, for AR, there is no particularly defined business model that can work long-term. It is also very early to evaluate the profitability of an AR-based business because the technology is still in its development stages. Further, because of the lack of AR development and application design standards, AR technology faces a problem relating to compatibility with the overall scenario. Security and privacy are also major concerns in the AR industry. Poor content quality, in addition to some technical software and hardware limitations in each game design, is an ongoing challenge in AR gaming. For specific surgeries and in the medical field, accuracy is of prime importance because it is essential for surgeons to have tangible information on how and when the technology is used [101]. For fashion retail, scant research exists on AR, and its impact on the industry has not yet been realized significantly. Hence, many brands still hesitate to invest in AR.

**Fig. 16:**
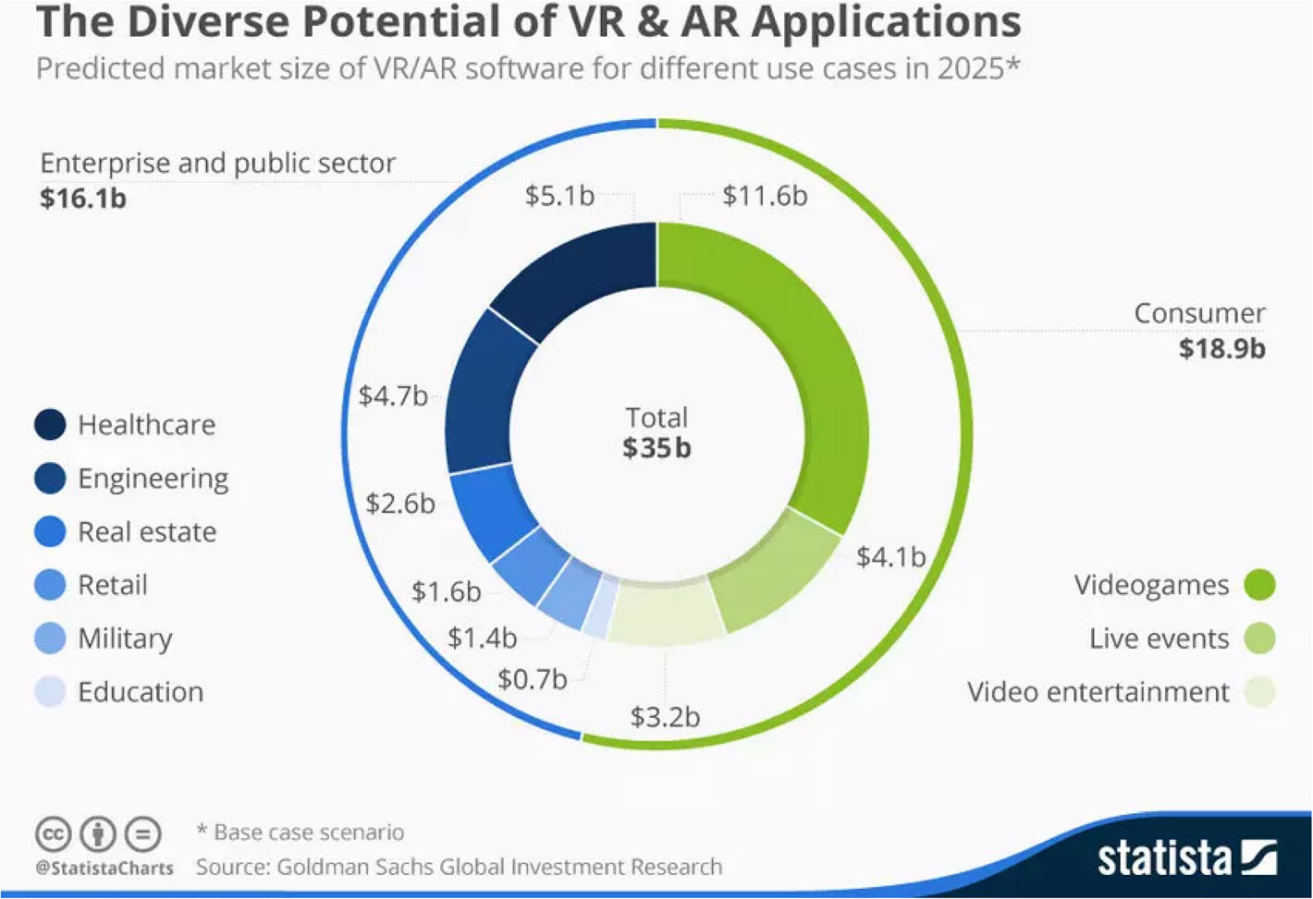
Estimated scenario of AR/VR in different sectors in 2025 (source: Goldman Sachs Global Investment Research, 2016)

Despite the numerous challenges, AR has an enormous scope in the near future to transform many industries. On overcoming the above mentioned obstacles, AR could have the power to revolutionize the entire market in every aspect. It has tremendous potential in areas, such as education, medicine, military, construction, automobile, travel, retail, art, and architecture [102]. AR is a futuristic technology that will change and reshape a number of business strategies developed by organizations. With increase in market competition, customers trust only companies who offer good quality products and extraordinary service. This means that many companies will prioritize incorporating AR, as it promises a personalized experience with products, which would attract more customers. It is also conjectured that the mobile AR technology, which will rise in the coming years, would lead to greater social acceptance. As many people are familiar with operating mobile phones, it would be easier for them to adapt to new technology. Further, as mentioned in the previous section, AR can be very helpful in the current COVID-19 crisis, as it would be, in similar situations that may arise in the future. Figure 16 represents the estimation of the projected AR/VR scenario in different sectors in 2025. However, this report was given by Goldman Sachs in 2016. Considering the present COVID-19 situation and the likely post-lockdown scenario, it appears that people will still be hesitant to use the entertainment, retail, or medical facilities freely. This necessitates the use of AR to provide a fully immersive experience to the customers in almost every field. Hence, it would be wise to say that the AR/VR estimation for 2025 could supersede Goldman Sachs’ 2016 prediction.

## Conclusion

AR provides unique entertainment options that are not available with common types of digital media. With new research, future AR systems are bound to be significantly more advanced, compared to the currently available ones. Owing to AR, interactivity and content quality are noticeably different, and personalization is possible. The technology is new, and despite having been around for a considerable amount of time, it has not been fully and functionally incorporated in day-to-day activities such as retail and medicine owing to concerns such as technology, social acceptance, and usability. However, upon overcoming these challenges, AR has the ability to redefine gaming through enhanced content in real time. The use of AR in medicine may change the way surgeries are performed. Medical training and post-surgical treatments can be performed with ease using AR displays. As consumers desire new innovations that may simplify shopping experiences and make them more comfortable, they are most likely to welcome AR with excitement. We have also studied the existing AR solutions that are being implemented and have discussed its importance to recovery from the pandemic. Hence, AR is playing a very important role in providing users with technology experience like never before in almost all areas.

The most recent inventions are proofs of the growing improvements in AR. AR in gaming can be seen in Pokémon Go, which also makes use of GPS, and is therefore a location-based application. Snapchat, on the other hand, is an example of a marker-based application, which uses image recognition in addition to AR. There are many AR-based software development kits and the factors determining the choice of an appropriate SDK include the cost, platforms, image recognition technology and the possibility of 3D tracking and recognition. Unity and AR toolkit are a few of the engines that can be used to create AR apps. Google and Android have also provided their respective kits, Google AR Core and AR Spark studio. The instances that have been discussed show the growing market base of AR systems and their importance in the market. Hence, the importance of a review that provides insight into three major fields where AR systems are being used cannot be overemphasized.

## Data Availability

All relevant data and material are presented in the main paper.

## Abbreviations

AR: Augmented reality
VR: Virtual reality
HMD: Head mounted displays
HUD: Head-up display
GPS: Global positioning system
EAR: Enhanced augmented reality
PARIS: Personal augmented reality immersive system
VRF: Virtual fitting rooms
IV: Integral video
NOTES: Natural Orifice Transluminal Endoscopic Surgery
AVM: Arteriovenous malformations
NI: Natural interaction
RGB: Red, Green, Blue
HSV: Hue, Saturation, value
TAM: Technology acceptance model
